# Recent Advances in Nanoformulations for Quercetin Delivery

**DOI:** 10.3390/pharmaceutics15061656

**Published:** 2023-06-05

**Authors:** Ekaterina-Michaela Tomou, Paraskevi Papakyriakopoulou, Elmina-Marina Saitani, Georgia Valsami, Natassa Pippa, Helen Skaltsa

**Affiliations:** 1 Section of Pharmacognosy & Chemistry of Natural Products, Department of Pharmacy, School of Health Sciences, National and Kapodistrian University of Athens, 15784 Athens, Greece; ktomou@pharm.uoa.gr; 2 Section of Pharmaceutical Technology, Department of Pharmacy, School of Health Sciences, National and Kapodistrian University of Athens, 15784 Athens, Greece; ppapakyr@pharm.uoa.gr (P.P.); elminasait@pharm.uoa.gr (E.-M.S.); valsami@pharm.uoa.gr (G.V.)

**Keywords:** quercetin, nanoformulations, drug-delivery systems, antioxidant, anti-inflammatory

## Abstract

Quercetin (QUE) is a flavonol that has recently received great attention from the research community due to its important pharmacological properties. However, QUE’s low solubility and extended first-pass metabolism limit its oral administration. This review aims to present the potential of various nanoformulations in the development of QUE dosage forms for bioavailability enhancement. Advanced drug delivery nanosystems can be used for more efficient encapsulation, targeting, and controlled release of QUE. An overview of the primary nanosystem categories, formulation processes, and characterization techniques are described. In particular, lipid-based nanocarriers, such as liposomes, nanostructured-lipid carries, and solid-lipid nanoparticles, are widely used to improve QUE’s oral absorption and targeting, increase its antioxidant activity, and ensure sustained release. Moreover, polymer-based nanocarriers exhibit unique properties for the improvement of the Absorption, Distribution, Metabolism, Excretion, and Toxicology (ADME(T)) profile. Namely, micelles and hydrogels composed of natural or synthetic polymers have been applied in QUE formulations. Furthermore, cyclodextrin, niosomes, and nanoemulsions are proposed as formulation alternatives for administration via different routes. This comprehensive review provides insight into the role of advanced drug delivery nanosystems for the formulation and delivery of QUE.

## 1. Introduction

Quercetin (QUE), a widely known flavonoid, has been extensively studied for its pharmacological properties, such as antioxidant, anti-inflammatory, and anticancer [1]. However, the physicochemical characteristics and properties of QUE (e.g., low solubility and bioavailability) significantly limit its clinical application [2]. Therefore, many studies have investigated different nanosystems to improve QUE’s bioavailability and efficacy [3,4,5,6,7]. These systems can be used to develop QUE dosage forms with enhanced bioavailability after oral and/or other potential routes of administration. Different types of advanced delivery systems are used for the encapsulation, targeting specific tissues, and controlled release of QUE.

Drug-delivery systems based on pharmaceutical nanotechnology are vehicles for the delivery and targeting of active pharmaceutical ingredients (APIs). These drug-delivery nanosystems are already marketed products, mainly anticancer medicines. Due to their size at nanoscale, they offer several advantages over the widely used solid and liquid dosage forms such as tablets, capsules, suspensions, and emulsions. These advantages are due to their physicochemical properties, especially their size and composed materials (i.e., lipids, surfactants, amphiphilic block copolymers, etc.) [8,9]. Most of them are biocompatible and suitable for the delivery of both hydrophobic and hydrophilic APIs with controlled release properties. They can also improve the Absorption, Distribution, Metabolism, Excretion, and Toxicology (ADME(T)) profile of the encapsulated APIs. Nanoformulations can be applied in several modes of administration, ranging from intravenous (i.v.) and intramuscular (i.m.) to the transdermal and nasal route [10,11,12]. The targeting and the overcoming of physical barriers are two of the most important advantages that nanocarriers offer [8,9]. However, there are some limitations and challenges in the design and development of nanoformulations. Several techniques are required for the complete characterization of these systems, rendering their development more expensive compared to the conventional ones. The cost of nanomaterials is also a challenge for the pharmaceutical industry, as well as the lack of a regulatory landscape and the limited knowledge towards the performance of nanomedicines in humans [8,9].

**Figure 1:**
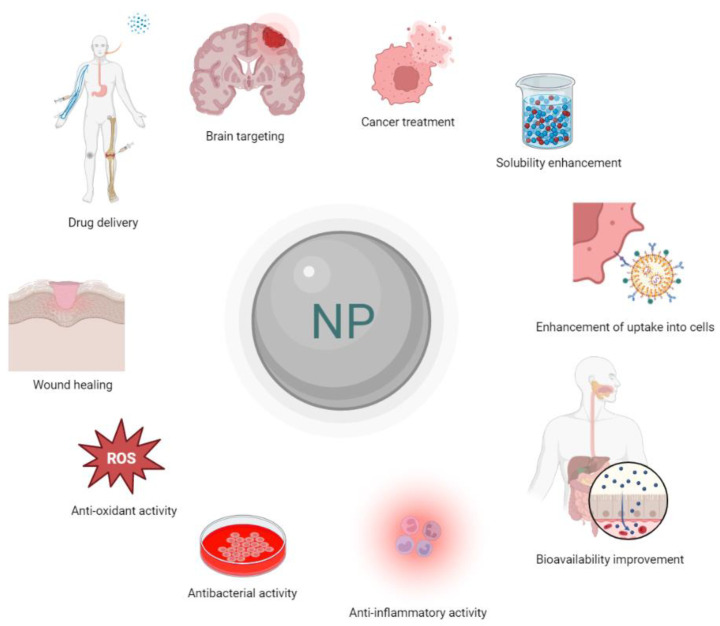
Illustration of nanoparticles’ (NP) applications in the field of pharmaceuticals.

**Figure 2:**
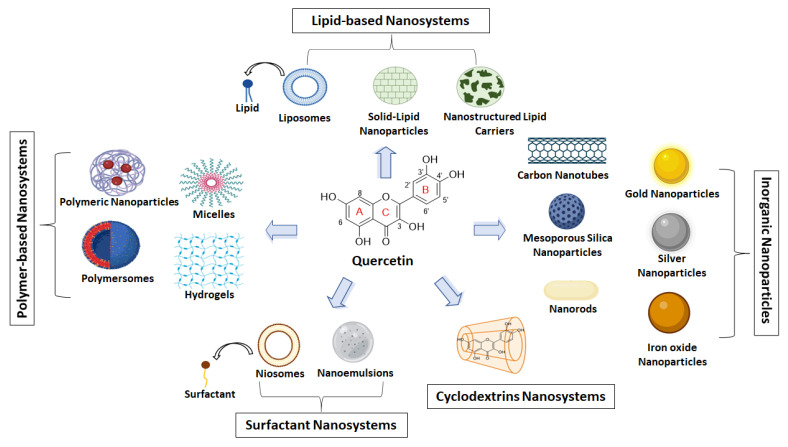
Illustration of QUE-delivery nanosystems. The chemical structure of QUE was designed using the ChemDraw^®^ v.16.0 software.

The aim of this review is to summarize all the nanoformulations that are used for the delivery and targeting of QUE. The main categories of drug delivery nanoformulations are illustrated in Figure 1, while in Figure 2, those used as QUE-delivery nanosystems are depicted. Their properties and applications were thoroughly explored and critically commented based on the literature in-vitro characterization experiments and preclinical data.

Each of the studied categories exhibits special characteristics and properties, ideal for controlled release, penetration to cells and/or tissues, and administration via alternative routes (i.e., transdermal or oral administration). Nevertheless, each category is accompanied by certain limitations and problems concerning the development of the final formulation. In Table 1, the advantages and disadvantages of nanoparticles are presented, rated as “high”, “medium”, and “low” importance, based on the results and conclusions of the original papers. Each class of nanoparticles exhibits unique characteristics that are useful for the loading, release, and targeting of APIs. Special attention is also given to the scale-up and regulatory issues that are of paramount importance for the development of nanoformulations and their translation into marketed products.

## 2. Materials and Methods

An extensive search was performed in the electronic databases PubMed, Scopus, and Google scholar for English-language publications. The publications from the past decade (2013–2023) served as a comprehensive guide for the systematic investigation of QUE’s nanoformulations. The article’s research was focused on experimental preclinical studies, including the development and evaluation of QUE nanosystems. The search was based on the following terms: quercetin and lipid-based nanocarriers, liposomes, nanostructured-lipid carries, solid-lipid nanoparticles, polymer-based nanocarriers, cyclodextrin, niosomes, and nanoemulsions. The authors performed the literature search excluding the studies with full text unavailable, publication language other than English, and conference abstracts. After the screening of titles and abstracts, 139 full-text studies were included in the part of quercetin nanoformulations.

## 3. Origin, Physicochemical Characteristics, Biosynthesis, and Main Pharmacological Activities of QUE

### 3.1. Origin, Physicochemical Characteristics, Pharmacokinetics, and Metabolism

QUE (3,3′,4′,5,7-pentahydroxyflavone), derived from the Latin word “quercetum” (oak forest), is one of the major flavonoids in the plant kingdom [13]. It is a principal component of the human diet, being widely presented mainly as glycosylated derivatives in vegetables (e.g., onions, garlic), fruits (e.g., apples, berries, cherries), and plant-based beverages (e.g., herbal teas, red wine) [14,15,16,17]. It is also reported that QUE represents approximately 60–75% of the total flavonoid intake [18].

QUE, a yellow-colored powder, is categorized into a class of flavonoids, namely 3-hydroxyflavones (flavonols) (Figure 2). Its molecular formula is C_15_H_10_O_7_, and its molecular weight (MW) is 302.24 g/mol [13]. The chemical structure of QUE contains hydroxyl groups at positions C-3 of the C-ring, C-5 and C-7 of the A-ring, and C-3′ and C-4′ of the B-ring, which determines the number of possible derivatives and has an important role in a molecule’s pharmacological activities [18]. Many QUE derivatives could be found, such as glycosides, methylated, and rarely occurring sulfate and prenyl substituents. QUE commonly occurs as a derivative in glycosidic forms mainly conjugated with glucose and rutinose. The most frequent glycosylation position is the hydroxyl group at C-3 [16,18]. In general, QUE is highly lipophilic and characterized by low solubility and bioavailability, with a half-life equal to 3.5 h [19]. Its water solubility is estimated to be 1 μg/mL, while in gastric and intestinal fluid, it is estimated to be equal to 5.5 μg/mL and 28.9 μg/mL, respectively [20]. However, the solubility varies among the QUE derivatives, and it is related to the type of substituents. For instance, its glycosylated forms could increase the molecule’s hydrophilicity [18]. QUE can be absorbed in the stomach, while the most important absorption site for QUE and its glycosides is the small intestine. The absorption process is mediated by the sodium-glucose cotransporter-1 (SGLT1) for glycosides or via passive diffusion for the aglycone [21]. Nevertheless, its bioavailability is considered extremely poor as only 20% of the orally administered dose can be absorbed. QUE’s bioavailability is significantly limited by the extensive intestinal and first-pass metabolism [22]. In Phase I, QUE is metabolized by CYT P450, while in Phase II, it is conjugated in the small intestine through the processes of glucuronidation, sulphuration, and methylation [23]. Then, the metabolites are secreted in the bloodstream and lymph circulation. The orally administered QUE is excreted via urine and feces, while a part of QUE metabolites is subjected to biliary excretion [24].

### 3.2. Biosynthesis

**Figure 3:**
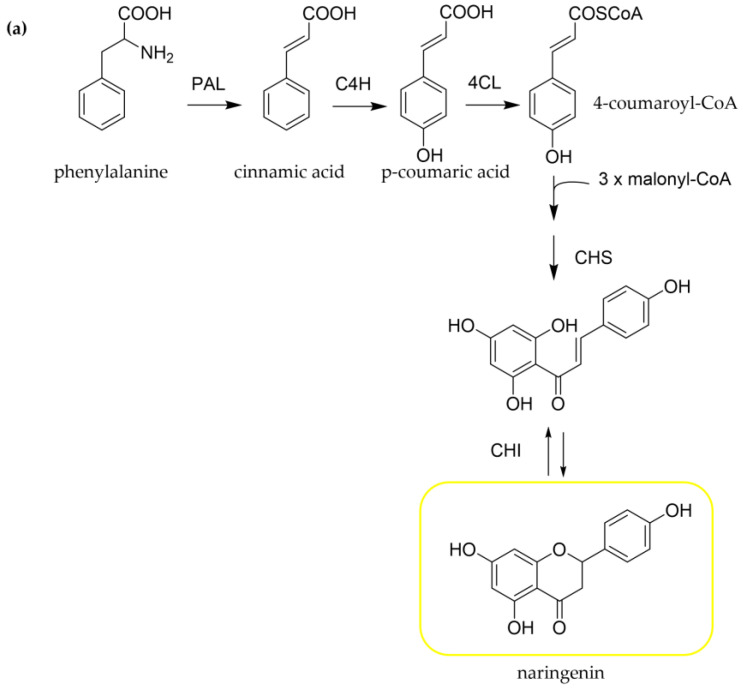

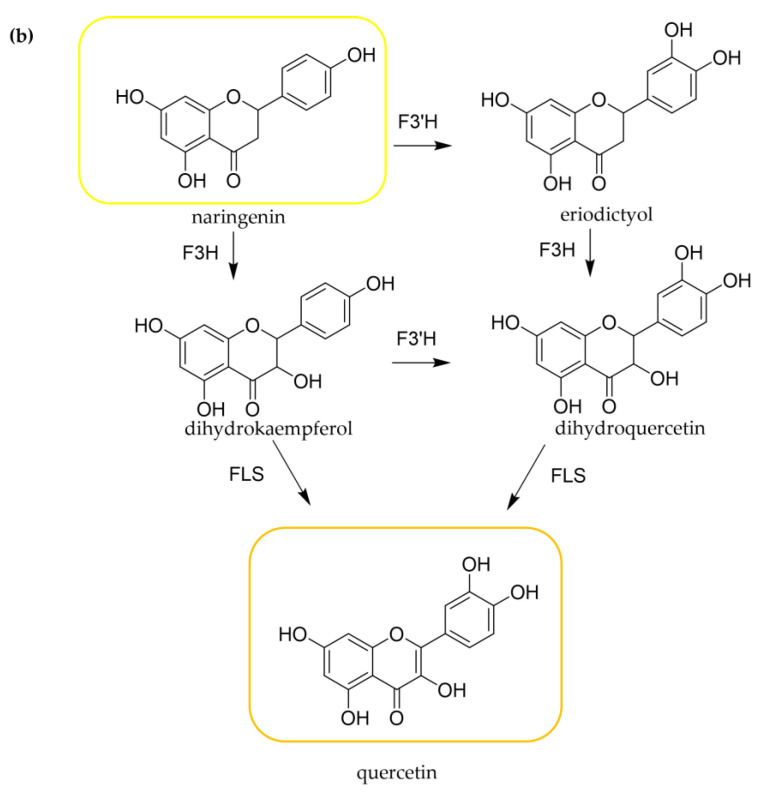
QUE biosynthetic pathway (ChemDraw^®^ v.16.0 software). (**a**) Schematic biosynthesis mechanism from L-phenylalanine to the flavanone, naringenin. Naringenin structure is marked with a yellow colour box. (**b**) Schematic biosynthesis mechanism from naringenin to QUE. Its structure is marked with an orange colour box. Abbreviations of the involved catalytic enzymes are shown.

Flavonoids are biosynthesized by a combination of the shikimate and the acetate pathway, involving several enzymes [25,26,27,28,29]. L-phenylalanine, a precursor of a broad range of natural products, is converted to cinnamic acid through the enzyme phenylalanine ammonia-lyase (PAL) (Figure 3a). Subsequently, p-coumaric acid (4-hydroxycinnamic acid) is formed by the hydroxylation of cinnamic acid via the enzyme cinnamate 4-hydroxylase (C4H) and finally produces 4-coumaroyl-CoA (4-hydroxycinnamoyl-CoA) by the addition of a co-enzyme A (CoA) unit with the involvement of the enzyme p-coumarate: CoA ligase (4-CL). This product, 4-coumaroyl-CoA (4-hydroxycinnamoyl-CoA), represents the beginning of the synthesis of specific flavonoids, which starts with chalcone formation. Specifically, one p-coumaroyl-CoA unit reacts with three molecules of malonyl-CoA, producing naringenin chalcone through the enzyme chalcone synthase (CHS). This reaction leads to the creation of A- and B-rings of the flavonoid skeleton. The heterocyclic C-ring is formed by chalcone isomerase (CHI) giving a flavanone, namely naringenin. From flavanones, different flavonoid structures such as flavones, flavonols, anthocyanidins, and catechins could be produced (Figure 3b). Modifications to the hydroxylation patterns in the two aromatic rings could be generally formed at the flavanone or dihydroflavonol stage [29]. Dihydroflavonols are produced by flavanone via the catalysis of flavanone 3-hydroxylase (F3H). Regarding QUE biosynthesis, eriodictyol could be produced by flavanone 3′-hydroxylase (F3′H) and be converted into dihydroquercetin through F3H and then given QUE via the enzyme flavonol synthase (FLS) [26]. In addition, naringenin could form dihydrokaempferol with the F3H, which could be transformed after some reactions into QUE [26,27,28].

### 3.3. Main Pharmacological Properties: Anti-Oxidant and Anti-Inflammatory Activities

Several studies have exhibited a wide range of QUE’s pharmacological properties, including antioxidant, anti-inflammatory, anticancer, antidiabetic, and antimicrobial [1,13,14,15,30]. Among them, the antioxidant and anti-inflammatory activities are of major importance since they are associated with the prevention and treatment of various diseases, such as cancer, cardiovascular diseases, and age-related disorders [13,31,32].

QUE has been unveiled as a potent antioxidant agent in many studies [14,31]. This activity depends upon the arrangement of functional groups in its structure (structure activity-relationships). Specifically, it is related to the presence of (i) The orthodihydroxy or 3′,4′-catechol group; (ii) The 3- and 5-hydroxyl groups; and (iii) The Δ2 double bond close to a 4-oxo group [16,18,32]. In general, QUE’s antioxidant mechanism is mainly assigned to its effects on glutathione (GSH), reactive oxygen species (ROS), signal transduction pathways, and enzyme activities [33,34]. GSH is considered one of the most abundant antioxidants, playing a critical role in protecting cells from oxidative damage and toxicity, as well as maintaining redox homeostasis [35,36]. Its expression levels are also associated with various pathological conditions [35]. Interestingly, it is reported that the C4-keto moiety of QUE and its Δ2 double bond facilitate the creation of its o-quinoid type metabolites [37,38] which could react with GSH, leading to reduced GSH content [37,39]. Gao et al. (2018) studied the effects of QUE on GSH metabolism, related enzymes, and signal pathways in rats [39]. They reported that 6-week treatment with 0.5% QUE supplemented polyphenol-free semisynthetic diet altered hepatic GSH metabolism by modulating GSH metabolic enzyme activities and mRNA expression. In addition, p38, extracellular signal-regulated kinase (ERK) 1/2 mitogen-activated protein kinases (MAPKs), and nuclear factor E2-like 2 (Nrf2) were involved in modulating GSH metabolism-related enzymes. Previous studies also mentioned the GSH reduction after long-term QUE administration in rats [37,40]. However, QUE could also act as a pro-oxidant agent depending on its concentration [37]. Furthermore, QUE is estimated as one of the most potent flavonoids for scavenging ROS [33,34]. As a result, it is also considered a good lipid peroxidation inhibitor, preventing cardiovascular and neurodegenerative diseases [13]. Hu et al. (2015) demonstrated that QUE reduced ROS production in acute gastric injury in mice, protecting H_2_O_2_-induced oxidative damage in gastric epithelial GES-1 cells [41]. In addition, QUE administration downregulated ROS production, such as H_2_O_2_, ROO, and HO, in a concentration-dependent manner in microglial cells [42]. It is also reported that QUE regulates various signal pathways [33]. For instance, it suppresses in vitro the oxidized LDL (oxLDL)-induced endothelial oxidative injuries by activating sirtuin 1 (SIRT1) and modulating the adenosine monophosphate-activated protein kinase (AMPK)/ nicotinamide adenine dinucleotide phosphate-oxidase (NADPH oxidase)/AKT/endothelial NO synthase-signaling pathway [43]. Moreover, QUE increased the mRNA expression of glutathione transferase (Gst) and aldo-keto reductase (Akr) involved in drug metabolism in an isoenzyme-specific manner, indicating its chemoprotective activity [44].

Many studies have investigated the anti-inflammatory activity of QUE [13,30]. It is generally reported that QUE inhibits in vitro, and in-vivo enzyme production involved in inflammation such as cyclooxygenase (COX) and lipoxygenase (LOX) [13]. Among other flavonoids, QUE inhibited both COX and LOX with stronger activity against LOX [45]. Furthermore, previous studies (in vitro and in vivo) showed that nitric oxide production and iNOS expression are repressed by QUE [15,46,47,48]. The inhibition of iNOS by QUE may be one of the responsible mechanisms for its anti-inflammatory activity [15]. Another mechanism of its anti-inflammatory effects seems to be the nuclear factor-kappa B (NF-κB) pathway downregulation [49]. QUE also inhibited the overproduction of tumor necrosis factor (TNF-α) and NO in lipopolysaccharide (LPS) stimulated macrophage in RAW 264.7 cell line [50]. Moreover, it was shown that QUE can inhibit LPS-induced mRNA levels of TNF-α and interleukin (IL)-1α in glial cells, resulting in decreased apoptotic neuronal cell death induced by microglial activation [51]. In addition, it is reported that QUE inhibits MAPK, activator protein 1 (AP-1), and NF-κB activity by inhibiting the c-Jun N terminal kinase and extracellular signal-regulated kinase [30]. Pretreatment of umbilical vein endothelial cells (HUVECs) with QUE showed its protective effects against H_2_O_2_-induced inflammation via the downregulation of vascular cell adhesion molecule 1 (VCAM-1) and CD80 expression [52].

## 4. QUE Nanosystems

### 4.1. Polymer-Based Nanosystems

Polymers are attractive tools for the pharmaceutical industry. These materials are derived from the connection of repeated monomers with high MW, resulting in a large chain structure [53]. Polymers that are formed by the linkage of two or more different types of monomers are called copolymers, and they have completely new properties. Those formed from the same monomer are called homopolymers. Polymer nanoparticulate platforms have been recognized as innovative delivery systems for the transport of APIs, nucleic acids, and other bioactive compounds [54]. A significant number of different configurations of polymer-based systems has been extensively described in the literature, including micelles, vehicles, core-shell structure, nanospheres, polymersomes, and hydrogels. In our research, we focused on the most recent literature about polymer-based formulations for the encapsulation of quercetin, which referred to various structures.

#### 4.1.1. Polymeric Micelles

Micelles are nano-scaled (less than 100 nm) colloidal NPs with a core-shell structure, consisting of a hydrophobic core and surrounded by a hydrophilic outer part [53]. Their formation is achieved spontaneously by the self-assembly of amphiphilic molecules in an aqueous solution. Their shape permits them to accommodate diverse molecules in their inner core. They can be used as delivery platforms for the transport of hydrophobic APIs and genetic material, while the hydrophilic shell contributes to the stabilization of the colloidal structure in a liquid medium [53,54].

Patel et al. (2022) developed QUE-incorporated micelles, which were composed of Pluronic P123 and Pluronic F88 [55]. The mixed micelles exhibited a mean particle size of approximately 22 nm and a slightly negative zeta-potential value of −16.2 mV, indicating a spherical structure with a very low critical micelle concentration. This formulation improved the bioavailability of QUE. UV–Vis spectroscopy was used to study the impact of polymer weight ratios and temperature on the solubilization of QUE. The findings revealed that as the temperature raised, the polymers’ thermal vibration increased, resulting in an expansion of the available space for QUE solubilization. Furthermore, the in vitro release kinetics of the compound from the mixed micelles displayed a distinct profile characterized by a slower and sustained release pattern. It was also shown that the QUE-loaded micelles exhibited significantly higher resistance to oxidation and a greater ability to inhibit the growth of MCF-7 tumor cells compared to the pure QUE. Therefore, mixed micelles incorporating QUE may be considered a viable strategy to enhance QUE’s oral bioavailability, antioxidant activity, and cell viability [55].

Qi and coworkers (2022) suggested a highly promising QUE carrier through the creation of micelles using Soluplus, which is an amphiphilic graft copolymer. The QUE-loaded micelles exhibited a uniform population with an average particle size of approximately 55 nm and surface charge close to neutrality (−1.41 mV). An increase in the weight ratio of Soluplus to QUE resulted in a corresponding increase in the QUE encapsulation ratio with a maximum value, approaching 100%, observed at a weight ratio of 16:1. The obtained results provide evidence for the stability of the QUE-loaded system at temperatures of 4 °C, 25 °C, and 37 °C. The system is also able to maintain a QUE encapsulation efficiency (EE) of over 90% for a period of 9 months. This investigation demonstrated several advantages of QUE-loaded Soluplus micelles compared to free QUE. The prepared micelles were found to facilitate the uptake of QUE into cells and aid its transportation to intracellular lysosomes and mitochondria. Results derived from in-vivo studies showed their improved ability to shrink solid tumors and reduce adverse effects. Soluplus–QUE micelles possess a multi-faceted inhibitory effect on angiogenesis, making them promising candidates for the targeted delivery of QUE in cancer treatment [56].

An approach that has been suggested for the enhancement of QUE’s poor bioavailability is its incorporation into Soluplus polymeric micelles with the presence of Poloxamer 407. The optimum formulation achieved 7% drug loading and an excellent EE up to 95%. The micelles were found to be physically stable, with a uniform particle size of 80 nm and high negative values of zeta-potential. These characteristics make it capable of evading the reticuloendothelial system and presenting an extended half-life (t_1/2_), as well as enhancing the uptake from the intestine. In-vitro release studies showed a sustained release of the substance for up to 10 days. Finally, the in-vivo study performed in beagle dog animal model showed significantly enhanced absorption and an improved systemic bioavailability of QUE compared to the pure substance [3].

The combination of Soluplus, Vitamin E succinate with polyethylene glycol (PEG) and Poloxamer 407 in varying ratios for the formation of mixed polymeric micelles was investigated as a strategy for QUE encapsulation to treat Glioma. The optimum formulation exhibited a particle size of 107.16 ± 1.06 nm, a polydispersity index of 0.236 ± 0.053, and a zeta-potential value of −1.19 ± 0.024 mV. The micelles possess a spherical morphology and were capable of fully accommodating the QUE within their interior, presenting an EE of approximately 77%, whereas QUE was released at a controlled release rate from the micelles. The findings of in-vitro cell viability study indicated that the QUE–PMMs had a considerably more effective cytotoxic profile, higher cellular uptake, migration, and induced apoptosis against C6 and U87MG cells compared to the pristine compound [57].

#### 4.1.2. Polymeric Nanoparticles

NPs can be composed of different biomaterials, such as lipids, polymers, etc. Polymeric NPs have largely attracted the interest of the scientific community [58]. They are distinguished from other particles due to their size, ranging from 10 nm to 10 μm [54], and their unique physicochemical properties [58]. NPs can accommodate many bioactive compounds (proteins, APIs, genetic material) into the inner part or in their exterior surface [59]. The achievement of a sustained molecule release in combination with NPs’ small dimensions permits them to transfer the compound to the targeted site of action. The controlled release can be achieved by modifying the external stimuli such as pH, temperature, irradiation, etc. [59].

pH-sensitive NPs based on Eudragit^®^ S100 polymer were developed by Sunoqrot et al. (2019) for the targeted delivery of QUE to colon cancer cells [60]. The researchers created various compositions by employing different weight ratios of the components. The most optimum one contained approximately 2.2% *w*/*w* of QUE in the crystalline state, and it included particles with an average size of 66.8 nm. It was characterized by slightly negative values of electrical potential and a moderate EE equal to 41.8%. It exhibited delayed release of the flavonoid in conditions mimicking the stomach and gastrointestinal tract environment while achieving a controlled release at pH~7. The evaluation of its cytotoxic profile on CT26 murine colon cancer cells indicated that QUE incorporated into NPs appeared higher antitumor potency than the free molecule [60].

**Figure 4:**
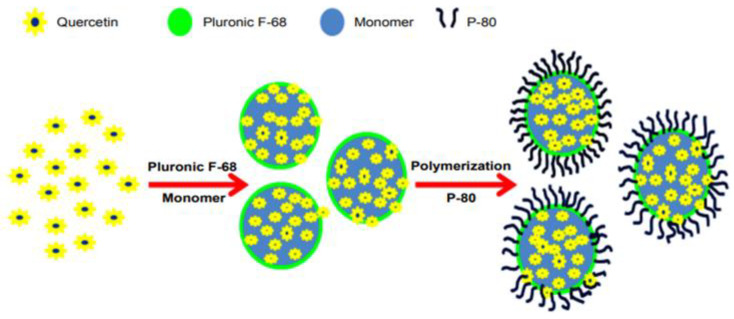
Schematic illustration of encapsulation of QUE-poly(n-butylcyanoacrylate) NPs by emulsion polymerization. Adopted from Bagad and Khan (2015) [61].

Another research work indicated the potential of poly(n-butylcyanoacrylate) (PBCA) NPs with or without polysorbate 80 (P-80) coating (QUE-PBCA+P-80 and QUE-PBCA, respectively) (Figure 4). The particle size of the aforementioned compositions was around 160–165 nm, whereas it did not occur noteworthy change after the addition of P-80. Their configuration was spherical with a smooth exterior surface, and some aggregations were observed in TEM images. Both NPs showed an efficient EE (74–79%), characterized by a 2-stage in-vitro release profile, as well as a burst release at the beginning followed by a controlled release pattern, resulting in higher bioavailability of QUE and improved pharmacokinetic parameters (AUC, C_max_, t_1/2_, and MRT values) [61].

Huang et al. (2022) developed a delivery platform for QUE using polymeric NPs composed of PEG conjugated with polyethyleneimine (PEI) polymers [62]. The NPS had an average size of approximately 21 nm. In-vivo studies using a mouse model with acute kidney injury were performed. The results proved that the changes observed in serum levels of critical biomarkers could be effectively controlled by the QUE polymeric PEG–PEI NPs. Moreover, this composition was capable of decreasing inflammation and oxidative stress, as well as restraining the renal degradation that occurred by acute kidney injury [62].

Another research work indicated the potential of polymeric QUE-NPs, consisting of poly(lactic-co-glycolic acid) (PLGA) and D-α-tocopherol polyethylene glycol 1000 succinate (TPGS), as an innovative therapeutic approach for triple-negative breast cancer. The homogenous, spherical population of NPs had a particle size of approximately 198 nm and a zeta potential value of −22.5 ± 2.5 mV. The EE and drug loading were determined to be 82.3% and 8.1%, respectively, resulting in a sustained release rate of QUE that was superior to that of the free substance. The pharmacological analysis revealed significant potential for the PLGA–TPGS QUE NPs in terms of their antitumor and antimetastatic effects [63].

#### 4.1.3. Hydrogels

Hydrogels are another category of polymeric formulations that can be used as delivery systems in the case of QUE. They are divided into natural, synthetic, and hybrids based on their origin. Their three-dimensional configuration is achieved by the cross-linking of polymers resulting in the capability to absorb high amounts of aqueous solutions into their polymeric network [64]. These multifunctional systems are ideal platforms for the sustained release of the encapsulated bioactives due to their high physicochemical stability, response to external stimuli, high loading efficiency, biocompatibility [65], biodegradability, and superabsorbency [54] that these systems ensure.

Hydrogels based on karaya gum-g-poly(acrylic acid) were developed by Bashir et al. (2018) as potent delivery platforms for the hydrophobic drug QUE [66]. The hydrogels exhibited porous configuration and resistance to mechanical stress. Their swelling properties differed in water and buffer solutions, presenting a better profile in acid than in neutral pH conditions. Up to 88% of the drug encapsulation was reached. In-vitro drug release studies revealed that pH values of medium equal to 7.4 had a beneficial effect on the QUE release from hydrogel’s network, achieving a percentage of 83% [66].

The production of a hybrid hydrogel by blending sodium alginate and poly(vinyl) alcohol (PVA) in different weight ratios was achieved by Esposito et al. (2020) [67]. The presence of micropores may be useful for the controlled release of the compound from the polymeric network. The hydrogels appeared to be non-thixotropic, pseudoplastic, and resistant to deformation, maintaining their antioxidant activity. QUE hydrogel with sodium alginate:PVA ratio equal to 2:1 was the optimum one since it presented excellent viscosity behavior, the highest swelling rate, and the most effective penetration during the ex-vivo skin models [67].

Mok and coworkers proposed a QUE delivery system consisting of a methoxy-poly(ethylene glycol)-l-poly(alanine) (mPEG-PA) polymer to prevent pain and delay the progression of osteoarthritis. After three days of incubation with mPEG-PA hydrogel, the viability and proliferation of human chondrocyte cells were assessed using a cell viability assay. The results of in-vitro release studies indicated the extended-release of QUE for approximately one month. The intra-articular injection of free-QUE hydrogel, QUE-loaded hydrogel, or saline solution in 12-weeks rats, in different doses, resulted in the reduction of cartilage degradation that occurred in osteoarthritis. The reduction was observed either with the free-QUE hydrogel or with the QUE-loaded one, while the presence of QUE in the formulation was found to provide additional protection against the disease. Thus, the proposed mPEG-PA hydrogel is a promising formulation for the intra-articular administration of QUE to control osteoarthritis [68].

#### 4.1.4. Polymersomes

Polymersome is a complex composition with a characteristic “pseudo-spherical” shell produced by the self-assembly behavior of amphiphilic block copolymers. Polymersomes’ construction is critically affected by some factors, such as the hydrophilic/hydrophobic ratio of a block copolymer. Their structure permits them to accommodate hydrophilic compounds into their aqueous interior, as well as to interact with the lipophilic molecules on their exterior membranes [54].

Sajadi and Khoee developed reversible stimuli-responsive polymersomes after synthetizing the β-(azobenzene-grafted dextran)-β-((methyl methacrylate)-r-(mono-methacrylate modified β-cyclodextrin)-r-(porphyrin acrylate)) amphiphilic block copolymer. The polymersomes maintained their self-assembly ability after UV irradiation and exhibited vesicular morphology. TEM and DLS analysis showed that the particle population had an average diameter of around 200 nm and the presence of some aggregations. Upon exposure to UV irradiation, the particle size increased to 250–300 nm, and fewer aggregations were observed. QUE was utilized as a drug model in a weight ratio of 1:10. Drug loading and EE values equal to 4.83% and 53.1% were reached, respectively [69].

Another research, focused on the encapsulation of polyphenols, such as QUE, into polymersomes, was performed by Gomes et al. (2018) [70]. Synthetic amphiphilic block copolymers consisted of hydrophilic monomers, such as acrylic acid or methacrylic acid, were combined with hydrophobic moieties and self-assembled to form these polymersomes. Subsequently, they confirmed the system’s colloidal stability and its capability to be used as drug-delivery system for polyphenols encapsulation and delivery [70].

### 4.2. Lipid-Based Nanoparticles

Due to their cell membrane-like structure, simplicity of surface modification, and high EE, lipid-based NPs have become widely used in administration via several routes. In particular, their applicability has been demonstrated in the delivery of low molecular-weight APIs, as well as in the field of gene transfer. In this section, the liposomal forms of QUE, as well as liposome-based NPs of the molecule i.e., polymer-grafted liposomes, phytosomes, etc., are presented. Solid lipid nanoparticles (SLNs) and Nanostructured Lipid Carriers (NLC) will also be discussed for their properties, especially in the oral administration of QUE.

#### 4.2.1. Liposomes and Liposome-Based Nanoparticles

Liposomes are vesicular systems composed mainly of phospholipids. The structural modification of the lipid bilayer is widely used to ameliorate their physical properties and extend the release profile of the encapsulated APIs. Modification of liposome surfaces using other biomaterials, i.e., polysaccharides, leads to different structural characteristics and biological behavior, as well as to a variable release profile of the encapsulated APIs [7,8,9].

QUE liposomes have been already evaluated for their efficacy in solid tumors in murine models and their protection against radiation-induced acute pneumonitis and late fibrosis by reducing oxidative damage [71,72].

QUE was formulated into “twin” liposomes. Namely, QUE and laccase (an enzyme) were successfully incorporated into liposomes using magnetoporation, which involves poking holes in the surface/membrane of liposomes. The laccase- and QUE-containing liposomes were linked and filled the pores with pH-sensitive components. It was discovered that this technique enhanced the release of QUE, as well as of laccase at an acidic pH and induced the death of tumor cells [73]. QUE liposomes prepared by a green thin-film dispersion protocol exhibited stronger anti-allergic effects in RBL-2H3 cells, including the decreased release of β-hexosaminidase and histamine, calcium influx, and the expression of inflammatory factors [2]. Diabetic nephropathy-affected rats received either a placebo, free QUE, PEGylated liposomes without API, or PEGylated QUE liposomes for a duration of two months. The liposomes were composed of soybean lecithin, cholesterol, and PEG 4000. The biochemical/pathological abnormalities in diabetic nephropathy were found to be improved by the liposomal form of QUE. Additionally, the liposomal form resulted in a higher concentration of QUE in plasma [74].

Phytosomes^®^ are considered as new delivery systems, based on food grade lecithin, for the increase of the solubility and bioavailability of APIs and vitamins and are widely used as food supplement delivery platforms. When given orally to human volunteers, a novel formulation composed of QUE Phytosome^®^ in film-coated tablets was found to significantly increase the bioavailability of QUE (10–20 times). QUE Phytosome^®^ has been shown to produce significantly higher plasma levels of QUE without causing any side effects. This increase is estimated to be up to 20 times more than other formulations and encapsulation processes [4]. Galactosylated chitosan was electrostatically adsorbed to the liposome surfaces composed of lecithin and cholesterol for targeting the liver’s asialoglycoprotein receptor (ASGPR). The outcomes demonstrated that mice’s livers efficiently acquired galactosylated chitosan-modified QUE-loaded liposomes following tail vein injection with a prolonged QUE release and enhanced macrophage M2 polarization. Additionally, it maintained low levels of liver enzymes and high levels of glutathione and lowered lipid oxidation in an acute liver injury [75].

In another study, QUE was encapsulated in folic acid-modified liposomes and proposed as a second-line treatment for osteosarcoma, a malignant solid tumor that primarily affects teenagers between the ages of 15 and 19. The liposomes were composed of QUE, lecithin, cholesterol, polyethylene glycol, and folic acid and were prepared via the thin-film hydration method accompanied by membrane extrusion for size reduction. The osteosarcoma, proliferation, and immune escape through the JAK2–STAT3–PD–L1-signaling axis can all be inhibited by the liposomal form of QUE by inhibiting JAK2 through the JH2 domain in a non-covalent way [76].

For intestine delivery, QUE was added to liposomes coated with Eudragit. The physicochemical properties of the liposomes with the Eudragit coating exhibited a variety of structures from small, spherical, uni-, and bi-lamellar to multi-lamellar liposomes. Additionally, the biological stability of the liposomes in fluids mimicking the gastrointestinal environment was enhanced by the Eudragit coating. By lowering the generation of reactive oxygen species and incorporating QUE into the vesicles, the human intestinal cells received the highest protection against oxidative stress [77].

The study of Bonechi et al. (2018), examined the synthesis of QUE- or rutin-loaded anionic, cationic, and zwitterionic liposomes prepared with freeze-thaw technique accompanied by the extrusion for size reduction. Their cytotoxicity and antioxidant properties were also evaluated. The Neutral Red Uptake test was used to measure the behavior of the prepared formulations in vitro. The prepared liposomes were around 120 nm with QUE of approximately 12% for all the liposomal formulations, which were found to be stable for three months. It was demonstrated that the cytotoxicity of rutin and QUE in zwitterionic and anionic liposomes was higher than that of their solution formulation. However, the harmful effects of empty liposomes could be inhibited by QUE and rutin contained in cationic liposomes [78].

The comparative biological effects of *Polygonum aviculare* L. herba (PAH) extract and QUE-entrapped liposomes on doxorubicin (Doxo)-induced toxicity in HUVECs, were also reported in the literature. Liposomes loaded with PAH extract and QUE (two liposomal forms prepared with phosphatidylcholine and with phosphatidylserine) were used to treat HUVECs. Controlled release profiles were also observed. The free and liposomal form of QUE inhibited oxidative stress and inflammation and reduced apoptosis, particularly in the form of Dipalmitoylphosphatidylcholine (DPPC) liposomes. The loading of QUE in liposomes increased cell viability and exerted better endothelial protection compared to free QUE, especially DPPC liposomes [79].

QUE–iron complexes have low solubility/hydrophobic properties that limit their pharmaceutical applications. To increase their bioavailability and therapeutic effect, these complexes are incorporated into liposomes. The interactions of QUE–iron complexes with dimyristoylphosphatidylcholine (DMPC) or palmitoyl-oleoyl phosphatidylethanolamine (POPE) multilamellar liposomes were studied, and the results showed that the complexes did not interact with the liposomes. QUE was able to penetrate lipid bilayers when added at a temperature higher than the main transition temperature of the phospholipids. The iron cations then entered the bilayers and formed complexes with QUE inside the liposomes. Heating the POPE liposomes improved the entry of the QUE–iron complexes. This approach can enhance the use of these complexes in medicine by integrating them into liposomes [80].

Another lipid-based delivery platform for QUE is nanocochleates. The development of nanocochleates showed a significant improvement in EE and controlled release of QUE for 24 h in comparison to liposomal formulations. Nanocochleates were composed of Dimyristoyl phosphatidyl glycerol (DMPG) and cholesterol (components of liposomes) using the trapping method. The nanocochleate formulation was more effective than liposomes on human mouth cancer cell lines and exhibited controlled release profile of QUE, probably due to their coiled structure [81].

Liposomal QUE formulations composed of lecithin were coated with lactose, chitosan, and inulin and evaluated for their potential radical scavenging activity. The coated liposomes exhibited pseudo-spherical and layered morphology and two particle populations at 200 nm (the primary one) and 600 nm. The pure lecithin liposomes (without polysaccharide coating) had higher antioxidant activity than the coated liposomal formulations, probably due to the chemical interactions between the QUE and coating polysaccharide. Among these three polysaccharides, lactose showed the best performance as a coating material, as its smaller size allowed for faster release profile of QUE in solution. Lactose coating was found to produce a better carrier for QUE, as it reduces its instability in the delivered media [82].

Long-circulating liposomes composed of soybean L-α-phosphatidylcholine and 1,2-distearoyl-sn-glycero-3-phosphoethanolamine-N-[methoxy(polyethylene glycol)-2000] with QUE were prepared by thin-film hydration and the extrusion method to be administered as treatment in ischemia/reperfusion injury. QUE-loaded liposomes, given in a rat model of liver damage, dramatically decreased inflammatory markers and enhanced healing, offering a viable treatment for this implication [83].

As mentioned above, QUE’s poor water solubility hinders its topical effectiveness. Three nanoformulations (liposomes, lipid nanocapsules, and smartCrystals^®^) were designed and developed to enhance QUE delivery to the skin. Liposomes were prepared via ethanol injection method by DPPC and Cremophor^®^ EL (polyoxyl 35 castor oil), lipid nanocapsules by lipophilic Labrafac^®^, Solutol^®^ HS 15, Cremophor^®^ EL, Lipoid^®^ S75-3, NaCl, and smart Crystals^®^ by the usage of Tween^®^ 80 or α tocopheryl polyethylene glycol 1000 succinate (TPGS) surfactants using a high-pressure homogenizer. The physicochemical characteristics of the formulations, the loading efficiency of the QUE as well as cellular interactions with keratinocytes and monocytes were evaluated and compared for the three formulations. Smart Crystals^®^ showed the highest loading efficiency, while the lipid NPs had the lowest size distribution. Lipid nanocapsules and smart Crystals^®^ were tested in vivo for delivery to human skin. No toxicity was observed in cell lines and efficient free radical scavenging ability was established. Skin penetration results showed that QUE smart Crystals^®^ deposited on the surface of the skin, suitable for a QUE-based sunscreen. Lipid nanocapsules allowed delivery to viable epidermis, promising for the treatment of skin inflammatory disorders such as psoriasis [84]. Furthermore, a mouthwash was developed that concurrently loaded with QUE and mint essential oil (mint oil) into phospholipid vesicles. The dispersion phase was made using a mixture of phosphate buffer, propylene glycol, and ethanol, whereas the bilayers were made using soy lecithin and Tween 80. *Streptococcus mutans* and *Lactobacillus acidophilus* growth was inhibited in the presence of the vesicles, showing their effectiveness [85].

Layer-by-layer self-assembly was used to create multilayered liposomes utilizing chitosan-oligosaccharides and N-succinyl-chitosan. The main goal was to improve the stability of L-α-phosphatidylcholine conventional liposomes and use multilayered liposomes as a transdermal drug delivery system with controlled release properties, boosting the penetration of QUE into the skin [86].

Another interesting formulation reported in the literature was that composed of pre-formed liposomes made of 2-distearoyl-sn-glycero-3-phosphocholine (DSPC) and cholesterol (CHOL), in a molar ratio of 55:45, and a liposome containing copper. The concentration of the flavonoid in the liposomes was above 5 mg/mL (resulting in 100-fold increase in solubility), and the QUE-to-lipid ratio was attained to be 0.2. The AUC_0–24h_ of this liposomal QUE was found to be equal to 8382.1 g h/mL (value acceptable for intravenous treatment in mice). The results after administration in mice indicated that the QUE was separated from the liposomal carrier [87].

In recent years, there is not any published data for the encapsulation of QUE into ethosomes and transethosomes, except for the study of Ferrara et al. (2022) [88]. Ethosomes and transfethosomes are lipid vesicular systems with ethanol at a concentration of around 20% for the increase of skin penetration. The presence of surfactants is also mandatory for the formation of transethosomes. Ferrara et al. (2022) prepared ethosomes and transethosomes with ideal physicochemical characteristics for the cutaneous delivery of QUE. Moreover, an increased uptake from human keratinocytes and melanoma cells, leading to enhanced cell migration; proliferation was also reported in the study. Transethosomes with the highest amount of phosphatidylcholine (PC) in their formulation were the best candidates for enhanced skin permeation [88].

#### 4.2.2. Solid Lipid Nanoparticles (SLNs) and Nanostructured Lipid Carriers (NLC)

SLN and NLC are lipid-based nanosystems composed of solid lipids, in most cases biocompatible and biodegradable. Several methods are used for their fabrication, but high-pressure homogenization is the most popular due to its speed and reproducibility. SLN and NLC are used for the encapsulation of hydrophobic APIs mainly for topical and oral administration [89,90,91].

QUE-loaded SLN with a negative charge was made via a coacervation method. Many physicochemical characterization methods were used to identify the particles, which were composed of arabic gum and stearic acid. The 26-h QUE release from the SLNs across a regulated exponential plateau pattern revealed a consistent distribution of the API inside the particles. The encapsulated QUE maintained a high level of antioxidant activity (81% of that of free QUE) compared to the non-encapsulated form [92].

Chitosan-coated QUE-loaded SLNs were prepared by ultrasonication method for the treatment of bladder cancer. The SLNs exhibited size around 250 nm, EE of QUE higher than 97% and sustained release approximately for 6 days. The cytotoxicity profile indicated that QUE’s toxicity was concentration-dependent, with an IC50 in the range of 1.6 to 8.9 g/mL [93].

The phase-inversion temperature method was used to make QUE-loaded SLNs utilizing a volatile oil—rosemary oil. Trilaurin solid lipid self-assembled into the smallest particle size with high stability. In SLNs produced with a rosemary oil-to-trilaurin ratio of 1:3, QUE could be loaded with an entrapment efficiency of over 60%. Polyoxyethylene-hydrogenated castor oil RH40 produced the particles with the smallest sizes depending on the concentration. In the QUE–trilaurin SLNs, which showed a prolonged biphasic release profile for more than 24 h, cell survival was greater than 75% [94].

**Figure 5:**
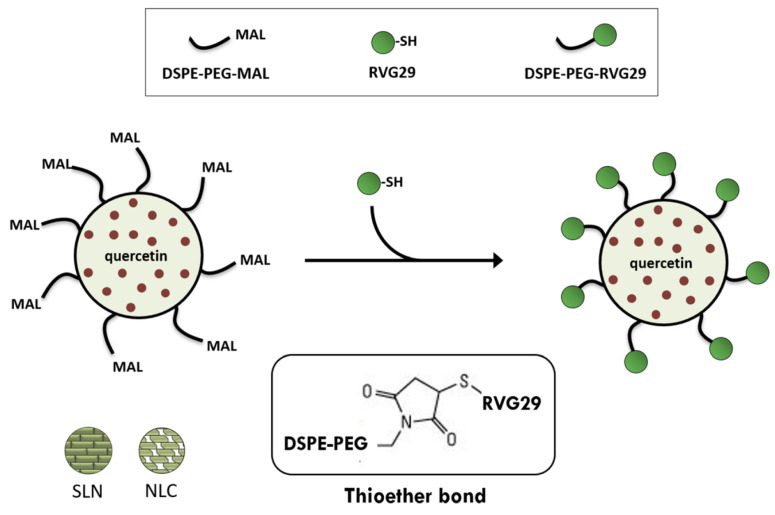
Schematic representation of nanoparticles functionalization with RVG29 peptide (not drawn to scale). DSPE-PEG-MAL is a 1,2-distearoyl-sn-glycero-3- phosphoethanolamine (DSPE) associated with polyethylene glycol (PEG) and terminal maleimide groups (MAL). The maleimide groups can react with thiol groups, thereby forming thioether bonds. Adopted by Pinheiro et al. (2020) [96].

SLNs and NLCs were prepared to evaluate QUE’s neuroprotective properties in Alzheimer’s disease. Both the lipid nanocarriers were functionalized with transferrin, which is overexpressed in brain endothelial cells, for enhancing the passage of BBB. The size of the prepared systems was around 250 nm, with a negative surface charge and EE higher than 80%. NLCs showed higher permeability across the BBB and the experiments on amyloid-beta revealed that NLC-transferrin could prevent the development of fibrils [95]. In the same context, RVG29–Functionalized SLNs and NLCs for QUE brain targeting in Alzheimer’s Disease were developed by hot homogenization and by sonication for size reduction (Figure 5) [96]. Furthermore, QUE was also loaded into SLNs utilizing tripalmitin and lecithin as the lipid core, while chitosan was applied as the coating material. QUE was compatible with the lipid core and polysaccharide coating. Moreover, the surface characteristics of the carriers increase the amount of SLN masking. In comparison to pure QUE powder, the release of flavonoid from the SLNs was more rapid at pH 7.0. Although QUE from the SLNs was more effectively absorbed by cells than the QUE from distilled water, the cellular uptake from the coated SLNs lagged behind that of the uncoated SLNs [97]. The SLNs were prepared using an emulsification and low-temperature solidification method to produce a formulation for the oral delivery of QUE. In animal experiments, SLNs are mostly absorbed passively into the ileum and colon. According to a pharmacokinetic study performed in rats, QUE SLNs formulation had a 571.4% higher relative bioavailability than QUE solution form [98]. To improve the effectiveness, erlotinib and QUE were injected into SLNs using chitosan–MA–TPGS polymer. The SLNs improved drug absorption in erlotinib-resistant cancer cells, reduced P-glycoprotein and nEGFR expression, and induced cell death. Increased lung tissue absorption and lower nEGFR expression were seen in SLNs in vivo. As a result, SLNs could be developed as a targeted medication for the treatment of non-small cell lung cancer being accompanied by few adverse effects [99].

Another study from the recent literature examined the synergistic effects of incorporating QUE with natural plant-oil-based NLCs for topical delivery of the drug to treat bacterial skin infections. Using various oils (sunflower, olive, corn, coconut, and castor), the study created five distinct NLC systems and examined their stability, structural characteristics, bioavailability, and antibacterial efficacy. The findings demonstrated the emergence of stable NLCs with great EE, an average size of less than 200 nm, and a zeta potential that was incredibly negative (−40 mV). QUE was encapsulated to boost the antibacterial activity and lessen the cytotoxicity of the systems against Staphylococcus aureus [100]. In the same context, NLCs were evaluated as a delivery system for piperine and QUE for oral squamous cell carcinoma treatment. The in-vivo experiments revealed that oral injection of the NLCs resulted in their effective distribution in the oral cavity [101].

For a fast evaluation of its anti-breast cancer activities, a phase inversion-based manufacturing strategy to produce biocompatible and biodegradable NLC loaded with QUE was also reported in the literature. The NLC particles demonstrated good stability, a sustained release pattern, and had EE and loading capacities of 95% and 11%, respectively. The solubility of QUE was multiplied by 1000 when NLCs were used. According to the findings of thermal analysis and spectroscopy, QUE was shown to be present in NLC in an encapsulated form. The NLCs formulation of QUE significantly boosted the cytotoxicity and dose-dependently induced death in breast cancer cells because of better QUE absorption [102].

The protective and antioxidant effects of QUE-loaded NLCs against toxicity induced by paraquat were also investigated. They were spherical, with a nano size range of around 50 nm and a high drug EE (higher than 95%). The results showed that NLCs were able to prevent paraquat-induced toxicity, including reactive oxygen species production and cell death, as well as restore mitochondrial membrane potential, lysosomal membrane integrity, and lipid peroxidation [103].

QUE-loaded cationic NLCs were created, and their distribution in vivo following oral administration was assessed. According to the findings, NLCs had an average particle size of around 130 nm, a very high EE of over 90%, and a positive zeta potential of approximately 40.5 mV. In vitro, the cationic NLCs were found to slowly release QUE, while when they were given to animals, higher AUC, and C_max_ values in the lung, liver, and kidney were observed compared to the control group. In particular, after their oral administration, the relative intake rates for the lung, liver, and kidney were 1.57, 1.51, and 1.68, respectively, compared to the QUE solution (control group) [104].

Piperine and QUE were co-encapsulated in NLCs. The solvent evaporation method, high shear homogenization, and sonication were used to formulate the NLCs, using also Compritol^®^ 888 ATO (solid lipid) and squalene (liquid lipid). The NLCs were around 200 nm with a negative surface charge, high EE, and drug release reaching 45% in just 12 h [105].

For the combination and targeted treatment of colorectal cancer, conatumumab, irinotecan prodrug, and QUE were co-incorporated in an NLC delivery system with over 70% uptake by HT-29 cells. The irinotecan prodrug’s release was accelerated in hypoxic conditions with sensitivity to reactive oxygen species. The multifunctional NLC demonstrated higher cytotoxicity when compared to free APIs [106].

QUE was co-encapsulated with α-tocopherol in NLCs containing chitosan or sodium alginate for wound healing applications. The NLCs were prepared by shea butter and argan oil, while the biopolymers used were given bio-adhesive properties. The size of NLCs was around 300 nm, with the surface charge strongly dependent on the used polysaccharide. The NLCs improved the cutaneous localization of QUE in injured skin regardless of the kind of bioadhesive polymer used, confirming their potential benefit for wound treatment [107]. Izza et al. (2022) also demonstrated that NLCs have successfully increased all antioxidant activity per unit concentration of polyphenols (specific antioxidant activity) in comparison to free polyphenols [108]. NLCs that included QUE exhibited better specific antioxidant activity than resveratrol or kaempferol, presenting the lowest EE [108]. In the same context, the combination of QUE and resveratrol produces synergistic effects and overcomes the other drawbacks of using single medicines to treat skin cancer. The application of central composite rotatable design revealed the ability of co-loading of these APIs in the optimized synthesis of NLC formulation to enhance the efficacy of the two entrapped polyphenols [109].

### 4.3. Surfactant-Based Nanoparticles

#### 4.3.1. Niosomes

Niosomes are microscopic vesicles composed of non-ionic surfactants of the alkyl or dialkyl polyglycerol and lipid compounds, such as cholesterol or L-α-soya phosphatidylcholine [110,111]. In aqueous media, non-ionic surfactants self-assemble to form a bilayer structure, with the hydrophilic heads pursuing contact with the solution and the hydrophobic tails oriented towards each other [110]. These movements of niosomes components result in the formation of an aqueous compartment, in the core of the structure, able to guest hydrophilic drugs, while the hydrophobic ones can be incorporated into the lipophilic bilayer [112]. Niosomes can be classified based on their size or the number of bilayers in their structure. According to these criteria, five categories can be described, such as multilamellar vesicles (MLV, 0.5–10 μm diameter), large unilamellar vesicles (LUV, 100–3000 nm diameter), small unilamellar vesicles (SUV, 10–100 nm diameter), bola-niosomes, and proniosomes. There are three established preparation methods: the slurry, the slow spray coating, and the coacervation phase separation method [113].

The in-vivo behavior of niosomes is comparable to the liposomal one, allowing the bioavailability and stability increase of the entrapped drug, as well as a more efficient organ distribution [110]. Niosomes are biocompatible, non-immunogenic, and biodegradable drug carriers that present several advantages arising from their structure and functionality. Particularly, they are considered appropriate for the encapsulation of low-solubility compounds or labile molecules, as their amphiphile nature favors a large variety of solubility along with the protection of the most vulnerable structural parts [114].

As already mentioned, QUE is a widely used dietary flavonoid proposed for the management of abundant indications, such as anti-inflammatory, antioxidant, cardioprotective, hepatoprotective, or anticancer agents [115,116,117,118,119]. Numerous niosome-based QUE formulations have been developed and evaluated in the literature, especially in the last five years, to serve as therapeutic agents for the aforementioned morbidities. Namely, in-vitro and in vivo studies on QUE niosomal systems have proven the enhancement of its antioxidant activity due to the higher stability and more efficient delivery [115,118,120]. Sadeghi–Ghadi et al. (2020), in their study, fabricated hyaluronic acid small niosomes (231.07 ± 8.39 nm) for QUE encapsulation, with high entrapment efficiency equal to 94.67 ± 1.62% [115]. The value of zeta potential (−34.00 ± 0.95 mV) indicates the stability of the produced niosomes, while they were characterized by adequate antioxidant activity. Further, in-vivo investigation in rats with carrageenan-induced paw oedema revealed the anti-inflammatory properties of QUE entrapped in polymeric niosomes [115]. In another study, the combinations of Tween 80/Span 80 and Tween 60/Span 60 with different additional components (polyethylene glycol derivatives, propylene glycol, etc.) were employed for the fabrication of antioxidant niosomes with potential applications in food and pharma. Among the tested formulations, the Tween 80/Span 80-based ones showed the smallest size (371–393 nm in the presence of PEG 400), while the maximum particle size was noted from Tween 60/Span 60-based systems containing PEG 10000 (around to 550 nm). When sonication treatment was added, the particle size values were lower in all cases, while the formulations with the minimum and maximum size were the same as without the sonication [120]. Elmowafy et al. (2020), in their study on sugar esters-based niosomes for QUE encapsulation, produced small niosomes (161 ± 4.6 nm) with high EE (83.6 ± 3.7%) [118]. The delivery systems were sustained-release formulations assessed for their in-vitro biocompatibility and drug-release profile. The system containing the Glucose C12 surfactant was selected as the most appropriate for the cells and in-vivo studies. The cell viability assay, along with the oxidative stress and histopathology experiments, showed the antioxidant and hepatoprotective effect of QUE niosomes when tested in HepG2 cells and rat animal model with CCl4-induced hepatotoxicity [118].

**Figure 6:**
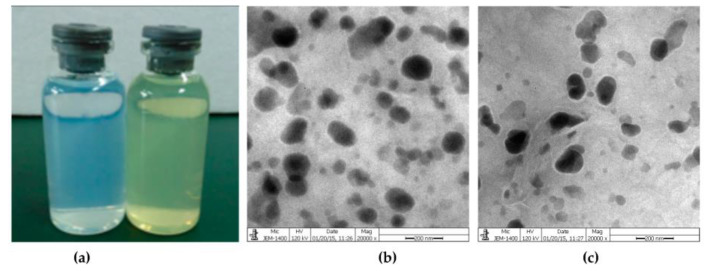
(**a**) Appearance of empty niosomes and quercetin-niosomes; (**b**) Transmission electron microscopy (TEM) image of blank niosomes; (**c**) TEM image of quercetin-niosomes. Adopted from Lu et al. (2019) [119].

The ability of niosomal nanocarriers to increase the skin penetration of the encapsulated QUE, as well as its antioxidant activity, was assessed in vitro and ex vivo using human skin. The fabricated niosomes were spherical Span60-RH40 systems of oval niosomes (Figure 6) of 97.6 ± 3.1 nm diameter and 31.1 ± 0.9 mV zeta potential range. Indicating their good stability. They manage to efficiently encapsulate QUE (87.3 ± 1.6%), increasing both the solubility and photostability of the flavonoid. The zeta potential of QUE-niosomes was lower compared to the empty ones, possibly due to QUE interaction with the surfactant’s head group that neutralized the negative charges on the niosomes’ surface. The ex-vivo experiments demonstrated that QUE was not able to penetrate across the dermis. However, the niosomes increase its penetration into the epidermis. Hence, this formulation was proposed as appropriate for cosmetics or the treatment of skin diseases [119]. In another study, highly stable with good EE Pluronic-based niosomes were developed for the delivery of doxorubicin and antioxidants (QUE and curcumin). The QUE-loaded vesicles presented a diameter of 437 ± 12 nm, while QUE combined with doxorubicin resulted in NPs of 380 ± 13 nm. Both formulations allowed the sustained release of the substance for over 24 h. Nevertheless, it was noted that a possible interaction between QUE and Pluronic decreases doxorubicin release from the system [116]. QUE has also been combined with captopril, in niosomes with particle size of 418.8 ± 4.21 nm. The hybrid formulation was characterized by sustained release and enhanced antihypertensive activity. Specifically, it manages to gradually decrease the systolic and diastolic blood pressure of hypertensive rats treated orally with 7.12 mg/kg of the formulation for 21 days. Moreover, lower levels of total cholesterol were reported after the treatment (−16.6%) [117].

#### 4.3.2. Nanoemulsions

Nanoemulsions are colloidal systems consisting of two immiscible phases (dispersed and continuous) with nanosized droplets dispersed in the continuous phase [121]. These systems are also called miniemulsions, fine-dispersed emulsions, or submicron emulsions [122], while different ranges for droplet size are reported in literature varying from 10 to 1000 nm [121,122,123,124], with 20–200 nm to be the most typical. Nanoemulsions can be either O/W (oil in water) or W/O (water in oil) systems, and the amount of oil in O/W usually varies between 5–20% *w*/*w* [125]. Moreover, double emulsions can be prepared; W/O/W (water in oil in water) and O/W/O (oil in water in oil), respectively [122], as well as bi-continuous nanoemulsions in which both the continuous and dispersed phases are liquid and stabilized by a surfactant. For nanoemulsion stabilization, three types of surfactants are used: nonionic, anionic, and cationic ones. Co-surfactants such as glycerin, ethylene glycol, propylene glycol, etc., can be added to reinforce the stabilization process [125]. Multiple methods of nanoemulsion preparation have been reported and can be classified into two general groups: high- and low-energy emulsification. The first one includes ultrasonic emulsification, high-pressure homogenization, and microfluidization, while the second one, phase inverts temperature and spontaneous emulsification [125].

Nanoemulsion delivery systems have been developed for several routes of administration, either for topical or systemic administration. In oral delivery, the increase of drug solubility, as well as the improvement of in-vitro dissolution, can enhance the absorption in the gastrointestinal (GI) tract [126]. W/O and O/W nanoemulsions are able to modulate these features of both hydrophilic and lipophilic molecules. Solubility enhancement may also enable the parental administration of insoluble drugs [127,128]. Furthermore, the sustained release of drugs from nanoemulsions, along with the ability of these systems to penetrate through membranes, renders them applicable in topical and ophthalmic delivery [125].

In three recent studies, O/W and W/O/W QUE-nanoemulsion carriers were fabricated by emulsification or emulsification-solvent evaporation method and assessed for their effect on breast and liver cancer cells [129,130,131]. In the case of O/W, the droplet size ranged from 21.7 to 26.4 nm [129,131], whereas in W/O/W, greater sizes were reported from 440 to 536 nm [130]. The experiments showed that the systems exhibit adequate cytotoxic and apoptotic activity on the cancer cells, due to the increase in the compound’s solubility [129,131]. Polysorbate 80-soy lecithin nanoemulsions enable targeted delivery [131] and sustained release of the entrapped compound, increasing QUE residence time on the tumor site and favoring the interaction with the DNA molecules [130,131]. Arbain et al. (2018), in their initial study, developed a QUE palm-based nanoemulsion applying the low- and high-energy emulsification method [132]. The mean droplet size of this system was 106 ± 0.44 nm, while the zeta potential was −43.7 ± 3.57 mV, indicating the stability of the system [132]. Further in-vitro evaluation of this nanoemulsion revealed the sustained release of the entrapped substance, as well as its selective cytotoxic activity against the A549 lung cancer cells [133]. Furthermore, QUE nanoemulsions have been proposed either as prophylactic agents against the side effects of chemotherapeutics [134], or for the improvement of the already established antitumor drugs via their synergistic interactions [135]. The protective activity is ascribed to the antioxidant properties of the flavonoid, which can scavenge and deactivate the ROS [134].

There is strong evidence of QUE protective activity against several metabolic diseases, such as hyperlipidemia, diabetes, and obesity, as well as in the cases of cardiovascular implications [136]. QUE nanoemulsions have been developed and tested in vivo for their therapeutic efficacy in high-fat-diet (HFD) rodents and streptozotocin-induced diabetic rats [137,138]. In these studies, the droplet size of the produced O/W and double layer O/W nanoemulsions ranged from 19.3 to 289 nm, and all three systems were characterized by good stability, as it was noted by the zeta potential values, which were lower or equal to −17.10 mV. The study of Mahadev et al. (2022) showed that the prepared nanoemulsion can increase QUE bioavailability and consequently amplify its antidiabetic activity [137]. Similar findings have been reported for a Tween 80-based nanoemulsion with a three-components oil phase (palm-based ester and lecithin), which can increase QUE solubility and protect against the diabetic induced cardiotoxicity [139]. Furthermore, the solubility enhancement achieved with the nanoemulsion formulation contributes essentially to GI absorption [139,140], as well as to the therapeutic effect of QUE, as it is depicted in the anti-obesity efficacy of an O/W nanoemulsion in HFD mice [139]. Higher efficacy was also argued by Son et al. (2019) after the treatment of HFD hypercholesterolemic rats with a nanoemulsion containing Captex^®^ 355, Tween 80, sodium alginate, and soy lecithin [138].

Nanoemulsions have been employed to increase skin QUE permeability, either for topical or systemic administration [141,142]. In particular, QUE nanoemulsions containing poly (lactic-co-glycolic acid) (PLGA) or hyaluronic acid (HA), with Tween-20 as emulsifier, have been assessed in ex-vivo permeation experiments using human cadaver skin. A chemical permeation enhancer was also incorporated into the synthesis resulting in twice higher permeability and better EE (≥87%) and particle-size distribution [143]. Hosny et al. (2021) combined QUE with isotretinoin in a self-nanoemulsion formulation aiming to reduce isotretinoin hepatotoxicity and increase the skin permeation of both substances [144]. The optimum formulation was characterized by 249-nm droplet sizes, consisting of rosehip oil, Lauroglycol-90 surfactant, and propylene glycol cosurfactant. In-vitro and ex-vivo permeation experiments confirmed the initial hypothesis proposing this formulation as a safe alternative for acne vulgaris [144].

QUE is considered an efficient weapon for the management of neurodegenerative [145,146] and autoimmune disorders [147,148] due to its anti-inflammatory properties. Recently, Alaqeel et al. (2022) fabricated a palm oil-based QUE nanoemulsion with lecithin and Tween 80, which was administered intraperitoneally (15 mg/kg/day for 30 days) in aluminum chloride (AlCl_3_)-induced Alzheimer’s disease rat model [149]. QUE administration improved the levels of all three measured neurotransmitters, dopamine, norepinephrine, and serotonin via the inhibition of enzymatic degradation, as well as the expression of inflammatory markers IL-1β, TNF-α, and adiponectin. However, the QUE nanoemulsion resulted in significantly better values of the measured biochemical parameters, revealing the contribution of formulation to flavonoid’s effectiveness [149]. In the study of Gokhale et al. (2019), a Carbopol-based nanoemulsion gel with arachis oil, Tween 20, and PEG 400 was developed for topical application in rheumatoid arthritis [150]. The formulation was characterized by adequate droplet size (136.8 ± 1.2 nm), good stability (−25.4 ± 1.7 mV, stable for 6 months), and excellent EE (94.65 ± 0.14%). The ex-vivo permeation studies showed that the nanoemulsion enables twice higher permeation than the free QUE at the time point of 24 h (62.51 ± 0.34% and 35.87 ± 0.21%, respectively). Moreover, the in-vivo experiments in a rat arthritic model revealed that QUE nanoemulsion treatment allows for the decrease of paw size and lower arthritic index. These two attributes are considered critical for the evaluation of disease progression [150].

### 4.4. Cyclodextrin-Based Nanoparticles

Cyclodextrins (CDs) are a group of cyclic oligosaccharides linked with α-1,4-glycosidic bonds, forming a structure of truncated cone with a hydrophobic inner cavity and hydrophilic outer surface. They can form stable inclusion complexes with several organic or inorganic lipophilic molecules, also enabling an adequate interaction of the outer hydroxy groups with water molecules or hydrophilic moieties [151]. CDs are widely used in pharmaceutical technology, with multiple applications in solubility and stability improvement, taste, and odor masking, as well as a decrease of toxicity [152]. Three natural CDs are mainly employed in pharmaceutical development, classified as α, β, and γ-CDs depending on the number of their glucopyranose units, while the hydroxypropylated and random methylated derivatives are the most long-established chemically modified CDs [151]. In nanotechnology, the incorporation of CDs in the nanostructured particles has been applied in numerous categories, such as the magnetic, polymeric, lipid, and solid-lipid NPs, as well as in the newer types like mesoporous, gold, and silver ones [153]. QUE formulation in CD-based NPs mainly includes metallic or polymeric systems. Specifically, Fe_3_O_4_-CD NPs have been produced either with α- or with β-CD to be used as QUE nanocarriers [6,154]. As α-CD presents pH-responsivity, it can be used to achieve pH-selective release of the encapsulated drug in the acidic environment of the tumor tissue (pH 6.8) protecting the normal tissues (pH 7.4) [155]. In the study of Ghafelehbashi et al. (2019), the addition of citric acid in different steps of the fabrication process was proposed to stabilize the system and impede the aggregation [154]. The resulting NPs were characterized by a mean diameter ranging from 39.36 to 51.51 nm, and a zeta potential from −34.9 to 30.9. They also presented significant magnetic properties that enable more targeted delivery. Citric acid and α-CD interact to produce the organic layer, while QUE was trapped in the cavity of CD forming an energetically favorable complex able to be disassociated and release the encapsulated substance in acidic conditions [154]. Fe_3_O_4_-β-CD NPs loaded with QUE were produced by β-CD and pluronic F68 and assessed for their effect in a mouse model for epilepsy disorder at the doses of 25 and 50 mg/kg, given intraperitoneally for 10 days. The manufactured NPs were nearly spherical with a diameter <50 nm, and their administration in mice reduced both the seizures and the hippocampal neuronal loss. Moreover, astrocytes’ activation was noted after both doses. The anticonvulsant effect of QUE was found to be more efficiently expressed when formulated in β-CD metallic NPs, probably due to the enhancement of its solubility and bioavailability [154].

The derivatives of natural CDs have been also used to encapsulate QUE in nanostructure organizations with polymers such as chitosan or PEG 2000 [156,157]. Chitosan–sulfo–butyl–ether–β–CD NPs loaded with QUE, and with a size ranging from 250 to 400 nm, which was developed using the ionotropic gelation technique. The resulting NPs were stable, with good EE (90%) reaching 99% of the maximum solubility in 24h. Furthermore, they showed a significant antibacterial activity against *Escherichia coli* (*E. coli*) [156]. The combination of ginsenoside Rg3 with QUE, DSPE-mPEG2000, and modified amphiphilic β-CD led to a spherical organization of small dimensions (<110 nm) and almost neutral zeta potential (6 ± 1 mV), indicating potential implications for system stability. The loading capacity was not very high for both natural products (12% for Rg3 and 6% for QUE). The highest release was observed in acidic conditions, favoring the selective targeting of tumors, as it was also revealed by the in-vivo imaging experiments [157]. In the study of Peñalva et al. (2019), the main protein of raw link, casein, was combined with 2-hydroxypropyl-β-CD to form QUE NPs for the flavonoid’s bioavailability enhancement [158]. In particular, this formulation enhanced the QUE plasma levels, impeding its systemic metabolism and the interactions of the flavonoid with the intestinal efflux pumps. Hence, the higher and prolonged absorption of the compound was depicted in the plasma pharmacokinetic profiles of rats receiving a single dose of (25 mg/kg) the QUE nano-formulation compared to the QUE oral solution [158].

### 4.5. Inorganic Nanoparticles

Inorganic NPs are a wide category of highly stable organizations composed of magnetic or other inorganic material [159]. They are non-toxic and biocompatible systems with magnetic properties that enable their use as theranostics, combining diagnosis and treatment. The main nanomaterials used for their fabrication are gold and silver, the oxides of iron, cooper, and the dioxides of titanium and silicon [160]. The QUE formulation into inorganic NPs is used either for imaging or for therapeutic purposes, especially in the cases of cancers, neurological, or metabolic diseases [161,162,163,164]. This review mainly focuses on the QUE delivery systems and includes the studies that propose inorganic nano-formulations assessed in-vitro or at preclinical levels in different disease conditions.

The application of NPs in cancer has been extensively proposed as a feasible alternative that ensures targeted treatment, minimizing the risk of toxicity against healthy cells [165]. It has been found that QUE is involved in multiple pathways associated with cell apoptosis (caspases and Bcl-2 genes) and cell survival or cell proliferation (MAPKs and Akt) [166]. In the literature, gold NPs [166], as well as zinc [167] and copper oxide [168] NPs, have been produced and tested in cell lines or animal models for their activity against breast cancer. The fabricated NPs in these studies were of different shapes (spherical, tetragonal, rod), and they showed a varying particle size from 3 to 50 nm. They were characterized by adequate stability, without signs of systemic toxicity, ensuring more efficient tumor targeting compared to the free NPs. The cancer cell treatment with inorganic QUE NPs induces cell death in human breast cancer cell lines [166,167,168] more efficiently than pure QUE, while the gold ones can downregulate the PI3K/Akt-signaling pathway related with the cells’ survival [166]. The animal studies in mice and rats treated with QUE encapsulated either in zinc or copper oxide NPs showed the higher reduction of tumor size compared with free QUE, a fact that demonstrates the enhancement of its absorption using these carriers [167,168]. Zinc oxide NPs as carriers of QUE have been also used in the human metastatic ovarian cancer cell line. The expression of the main protein markers involved in cell apoptosis (Bax, Bcl2, p53, caspase 3) was found to be enhanced, while cancer cells survival significantly decreased. Zinc oxide QUE NPs manage to induce the apoptosis of ovarian cancer cells via ROS production in lower dose than free QUE, probably due to the contribution of Zn^2+^ ions in ROS generation [169].

The application of inorganic NPs in neuroscience is widely spread in both field of imaging and targeted delivery in the central nervous system. Their electrical and magnetic properties enable the monitoring of brain diseases using multiple imaging methods [170]. Furthermore, the systems of inorganic NPs present high permeability and small size appropriate for Blood–Brain–Barrier (BBB) penetration, while their tendency to accumulate in tissues can be beneficial in the long-term treatment of neurological diseases [171]. Three studies in the same laboratory from 2018 to 2022 assessed the effect of QUE encapsulated in superparamagnetic iron–oxide NPs on learning and memory improvement of diabetic rats. They also discuss the protective activity of the flavonoid in the possible toxicity by these carriers [7,172,173,174]. The fabricated NPs were spherical with a mean diameter ranging from 30 to 50 nm. The higher efficacy of the nanoparticulate formulation was revealed in the mRNA expression of NF-κB target genes (BACE1, AβPP, Bax, Bcl2, and TNF-α), as well as in the results of the behavioral and histological analysis performed in diabetic rats and in their isolated brain tissues, respectively [7,172]. The bioavailability of QUE was enhanced when formulated in superparamagnetic iron–oxide NPs, and the NF–κB pathway was downregulated, resulting in lower neural cell death. Moreover, the increase of BACE1, AβPP, and Bax/Bcl2 levels is a strong indication of formulation ability to reverse neuroinflammation and diabetes-induced memory impairment [7]. The better results in Morris water maze and passive avoidance tests after the QUE superparamagnetic iron–oxide NPs administration and the improvement of pancreas, liver, kidney, and brain tissues morphology [172]. The higher efficacy noted by the nano-formulation compared to the free QUE is attributed to the enhancement of the molecule’s bioavailability, enabling the more dynamic interaction of the flavonoid with proteins related to the apoptosis and MAPK pathway. Hence, the enforcement of signal transmission and plasticity results in better information storage and improvement of learning and memory.

In the study of Liu et al. (2019), gold–palladium QUE NPs were prepared and tested in the SH–SY5Y cell line and mouse brain slices [175]. The NPs were of concave cubic shape and a 62-nm average particle size. The systems rescued the cells from Aβ-induced damage and showed high permeability across the BBB, as was demonstrated by the flow cytometry analysis. Furthermore, the NPs were characterized as biocompatible, showing non-significant toxicity to all the vital and disease-related mice tissues [175].

The role of NPs, especially inorganic ones, in inflammation is considered equivocal. Despite being blamed for toxicity and triggering immunological responses, NPs are also being proposed as therapeutic agents for treating inflammatory conditions [176]. In particular, metal and metal–oxide NPs, such as gold–, silver–, iron–, or zinc–oxide nano-systems can impede inflammation cascade by blocking the cytokines, inactivating the free radicals and inhibiting the biochemical pathways of NF-κB and COX-2 [177]. Hence, the incorporation of QUE into these systems may result in synergistic expression of their common anti-inflammatory and antioxidant properties. In two recent studies, the combination of QUE with artemisia and curcumin in two different silver N systems is proposed for the management of inflammation [178,179]. Namely, the artemisia/QUE NPs ointment was tested in vivo as wound healing agent in mice infected with cutaneous leishmaniasis. The NPs had a mean diameter size of 113.9 nm, and they were characterized by stability (−23.89 mV zeta potential). The 21-day topical treatment led to faster wound healing and less intense inflammatory response, as depicted in the inflammatory cells number [178].

The efficacy of gold and silver QUE NPs in wound healing has been also reported by two additional studies [180,181]. In both cases, the formulations manage to increase the cell proliferation and cell migration, accelerating the wound healing. More specifically, the gold NPs facilitate the absorption of QUE, which interacts with the TGFb1 (transforming growth factor-beta), leading to fast-scar formation [180]. Furthermore, in the study of Badhwar et al. (2021), a synergistic effect was noted between QUE and silver NPs, leading to a higher rate of re-epithelialization [181]. This effect is reinforced by the sustained release of the formulation, which increases the retention time of the substance on the wound [181].

In the study of Kumawat et al. (2022), QUE was used to achieve the functionalization of silver curcumin NPs, forming a dual surface (corona), where organic and inorganic molecules co-exist [179]. These NPs had a hydrodynamic size of 32.71 nm; they were stable (−25.1 mV zeta potential) and tested on mouse RAW 264.7 macrophages. The MTT assay revealed a 200% increase in-cell viability after their treatment with the QUE-functionalized NPs. Furthermore, they showed a mild induction of ROS production, as demonstrated by the results of the DCFH–DA assay and high antioxidant activity, as revealed by the results of the ABTS assay [179]. Conversely, the formulation of QUE into iron–oxide NPs reduces the anti-radical activity of pure flavonoids but preserves its antioxidant and anti-inflammatory effect against the H_2_O_2_-induced cytotoxicity in PC12 cells [182]. Moreover, QUE inorganic NPs can be used as a therapy against bacterial inflammation from gram-negative strains. Particularly, the encapsulation of QUE into gold NPs resulted in spherical particles with a 30-nm mean particle size that can interact with the external membrane of E. coli causing the lysis of the membrane.

## 5. Conclusions

In this literature review study, we presented polymer-, lipid-, surfactant-, and cyclodextrin-based nanosystems and inorganic NPs for the encapsulation, delivery, controlled release, and targeting of QUE. Special attention was given to the physicochemical characteristics of these delivery nanosystems and the added value that they offered to QUE properties, i.e., increasing its solubility and bioavailability. Moreover, all the characteristics of the described nanosystems for the formulation of this flavonoid with several biomedical applications were summarized in Table 2, aiming to reveal uncharted scientific territory for future studies. To the best of the authors’ knowledge, there is no publication in the literature where similar doses of QUE are encapsulated in different nanoparticles, and a comparison of their efficacy is made. We found only one publication in which lipid-based nanoparticles are evaluated and compared for skin-penetration purposes [84]. On the other hand, most publications about SLNs and NLCs include comparative studies about the effectiveness of QUE. For this reason, we prepared Table 1 and Table 2, which can be used as key references for formulation scientists for the development of QUE for a special target, i.e., nasal-to-brain administration, therapeutic purposes, controlled release, etc.

We should point out that all the soft-matter nanosystems increased QUE solubility, which is of paramount importance for its use in pharmaceutical applications. QUE antioxidant activity is also improved by the encapsulation into the lipid- and surfactant-based nanosystems. Polymer-based nanosystems are ideal for QUE-controlled release, while inorganic NPs offer increased stability and theragnostic properties. The anti-tumor and cytostatic properties of QUE can also be improved by several types of nanoformulations. In conclusion, QUE is a natural product with numerous pharmacological properties, and its encapsulation in drug-delivery nanosystems makes it a potential nanomedicine with several advantages in preclinical research.

## Figures and Tables

**Table 1:** The advantages and the disadvantages of various nanoformulations for drug delivery.

	Polymer-Based Nanosystems				Lipid-Based Nanosystems		Surfactant-Based Nanosystems		Cyclodextrin-Based Nanoparticles	Inorganic Nanoparticles
	Polymeric Micelles	Polymeric Nanoparticles	Hydrogels	Polymersomes	Liposomes	SLN-NLC	Niosomes	Nanoemulsions		
Advantages										
Biocompatibility	++	++	++	++	+++	+++	++	+++	+++	+
Biodegradability	+	++	+++	++	++	++	++	++	+++	+
High Loading efficiency	++	++	+++	+++	++	+++	++	+++	++	+
Chemical versatility	+++	+++	+	+++	+	+	+	+	+	+
Physicochemical stability	+++	+++	+++	+++	+	++	++	++	++	+++
Controlled release properties	+++	+++	++	+++	++	++	++	++	++	++
Administration by different routes	++	++	+	++	+++	++	++	++	++	++
Stimuli responsiveness	+++	++	+++	++	+	+	+	+	+	+++
Improve of ADME(T) profile	+++	+++	+	++	++	++	++	++	++	+
Targeting	++	++	+	+	+	+	+	+	+	+++
Imaging	+	+	+	+	+	+	+	+	+	+++
Theragnostic	+	+	+	+	+	+	+	+	+	+++
Precision medicine	+	+	+	+	+++	+	+	+	+	++
Disadvantages										
Nanotoxicity	++	++	+	++	++	++	++	+	+	+++
High cost	+	+	+	++	++	++	++	+	+	+++
Limitations in scale-up	+	+	+++	++	++	++	++	+	++	+++
Immunogenicity	++	++	+	++	+	+	+	+	+	+++
Lack in regulatory framework	++	++	+++	+++	++	++	+++	+	+++	++

SLN-NLC: Solid lipid nanoparticles-Nanostructured Lipid Carriers; ADME(T): Absorption, Distribution, Metabolism, Excretion and Toxicology. High: +++, Medium: ++, Low: +.

**Table 2:** The added value of nanoformulations in improving the properties of QUE.

	Polymer-Based Nanosystems				Lipid-Based Nanosystems		Surfactant-Based Nanosystems		Cyclodextrin-Based Nanoparticles	Inorganic Nanoparticles
Properties of QUE	Polymeric Micelles	Polymeric Nanoparticles	Hydrogels	Polymersomes	Liposomes	SLN-NLC	Niosomes	Nanoemulsions		
Improved solubility	x	x	x	x	x	x	x	x	x	
Improved EE	x		x						x	
Co-loading with other APIs					x	x			x	x
Increased bioavailability	x		x		x	x			x	
Sustained release		x					x			
Controlled release		x	x		x				x	
pH-responsive release							x			
Increased stability				x						x
Targeting to tumors		x			x			x	x	x
Targeting to brain						x				
Transdermal administration						x	x			
Facilitation of the uptake into cells	x				x					x
Improved anti-inflammatory properties		x	x		x		x	x		x
Enhanced antioxidant activity	x					x	x	x	x	x

SLN-NLC: Solid lipid nanoparticles-Nanostructured Lipid Carriers.

## Data Availability

Not applicable.
